# Supplementary material for: Genetic Diversity for Agronomic Traits and Phytochemical Compounds in Coloured Naked Barley Lines
Source: Plants (Basel). 2021 Jul 30;10(8):1575. doi: 10.3390/plants10081575 (PMC8398654; doi:10.3390/plants10081575)
Supplement: Supplementary file 1 [file plants-10-01575-s001.zip › plants-1317051-supplementary.pdf]

Table S1. Mean, standard error, minimum and maximum values, range, coefficient of variation and least significant difference for the six agronomic traits of the 71 barley accessions grown at Foggia (Italy) during 2015-2016 growing season.

**Trait**	**Mean ±SE**[b]	**Min**	**Max**	**Range**	**CV (%)**	**LSD**$_{(P \leq 0.05)}$
Heading time (days)[a]	6.71 ± 0.21	1.33	11.33	10.0**	25.8	1.05
Plant height (cm)	109.2 ± 0.79	96.7	130.0	33.3**	6.1	6.20
Seed yield (t ha^{-1})	4.40 ± 0.09	2.82	6.61	3.8**	16.5	0.47
Specific weight (kg hL^{-1})	77.0 ± 0.33	64.5	81.9	17.4**	3.6	1.14
1000-seed weight (g)	37.7 ± 0.33	32.3	47.1	14.8***	7.4	6.50
Lodging (0-9)[c]	3.83 ± 0.30	0.00	8.00	8.00**	66.7	0.85

[a]from April 1.

[b]SE, standard error; CV, coefficient of variation; LSD, least significant difference.

[c]scale: 0 = very low value; 9 = very high value.

, * statistically significant: 1%, 1‰ level of probability, respectively.

Table S2. Analysis of variance (mean square values) for 5 agronomic traits for the 20 barley genotypes evaluated in 2017-2019.

Source of variation	Df[a]	HT (days)	PH (cm)	SY (t ha^{-1})	SW (kg hL^{-1})	TSW (g)
**Year (Y)**	2	284.60	190.17	44.72	90.94	160.84
**Genotype (G)**	19	17.89	286.05	1.78	77.88	51.38
**G × Y**	38	3.25	60.74	0.24	5.55	10.15
**Error**	114	0.49	12.29	0.09	0.61	1.14

[a]df, degrees of freedom; HT, days to heading, from April 1; PH, plant height; SY, seed yield; SW, specific weight; TSW, 1000-seed weight.

Genotype (G), Year (Y) and G×Y interactions were significant sources of variation at 0.0001 probability level, for all traits measured.

Table S3. Analysis of variance (mean square values) for 9 biochemical traits for the 20 barley genotypes evaluated in 2015-16.

Source of variation	df[a]	TPro	TCarot	β-glu	TPh	TPAnt	TAnt	TFlav	DPPH	TEAC
**Genotype (G)**	19	3.12	1.28	0.75	225338	70529	3665.9	99974	8.60	7.40
**Error**	38	0.02	0.05	0.03	2909	2268	12.9	13764	1.75	0.07

[a]df, degrees of freedom; TPro, total proteins (%); TCarot, total carotenoids (μg β carotene g^{-1} dry matter); β-glu, β-glucans (%);TPh, total polyphenols (μg gallic acid equivalents (GAE) g^{-1} dry matter); TPant, total proanthocyanidins (μg catechin equivalents (CE) g^{-1} dry matter); TAnt, total anthocyanins (μg CE g^{-1} dry matter); TFlav, total flavonoids (μg gallic acid g^{-1} dry matter); DPPH (μmol of Trolox equivalent for g^{-1} dry matter); TEAC (μmol of Trolox equivalent g^{-1} dry matter).

Genotype (G) was significant source of variation at 0.001 probability level, for all traits measured.

Table S4. Mean values for the 14 agronomic and biochemical traits according to the 3 clusters grouped from the cluster analysis of the 20 barley genotypes.

Trait[a]	I (11)[b]	II (7)	III (2)
**HT***	11.6b	12.4ab	**14.7a**
**PH*****	93.6b	**101.1a**	98.9ab
**SY**	4.7a	4.3a	5.0a
**SW*****	**80.4a**	**80.6a**	74.7b
**TSW***	37.8b	38.2ab	**40.9a**
**TPro*****	12.6b	**13.6a**	11.4c
**TCarot*****	2.1b	2.3b	**3.4a**
**β-glu*****	3.9b	**4.2a**	**4.5a**
**TPh**	2387.1a	2351.2a	2390.0a
**TPAnt*****	1309.6b	1310.7b	**1576.5a**
**TAnt*****	**101.2a**	72.7b	7.7c
**TFlav*****	**1140.0a**	890.0b	1027.8ab
**DPPH**	10.5a	10.5a	10.6a
**TEAC****	12.8b	**13.3a**	12.8ab

[a]HT, days to heading from April 1; PH, plant height (cm); SY, seed yield (t ha^{-1}); SW, specific weight (kg hL^{-1}); TSW, 1000-seed weight (g); TPro, total proteins (%); TCarot, total carotenoids (μg β carotene g^{-1} dry matter); β-glu, β-glucans (%);TPh, total polyphenols (μg gallic acid equivalents (GAE) g^{-1} dry matter); TPant, total proanthocyanidins (μg catechin equivalents (CE) g^{-1} dry matter); TAnt, total anthocyanins (μg CE g^{-1} dry matter); TFlav, total flavonoids (μg gallic acid g^{-1} dry matter); DPPH (μmol of Trolox equivalent g^{-1} dry matter); TEAC (μmol of Trolox equivalent g^{-1} dry matter).

[b]No. of genotypes.

*, ** and *** represent 0.05, 0.01 and 0.001 significant differences among clusters, respectively.

Data with different letters within rows indicate significant differences (Tukey-Kramer tests).

Data in **bold text** indicate highest value(s) within each row (i.e., trait) across the 3 clusters.
